# Overactivation of Intestinal SREBP2 in Mice Increases Serum Cholesterol

**DOI:** 10.1371/journal.pone.0084221

**Published:** 2014-01-20

**Authors:** Ke Ma, Pooja Malhotra, Vinay Soni, Omar Hedroug, Fadi Annaba, Amish Dudeja, Le Shen, Jerrold R. Turner, Ekaterina A. Khramtsova, Seema Saksena, Pradeep K. Dudeja, Ravinder K. Gill, Waddah A. Alrefai

**Affiliations:** 1 Research and Development, Jesse Brown VA Medical Center, Chicago, Illinois, United States of America; 2 Division of Gastroenterology and Hepatology, Department of Medicine, University of Illinois at Chicago, Chicago, Illinois, United States of America; 3 Department of Surgery, University of Chicago, Chicago, Illinois, United States of America; 4 Department of Pathology, University of Chicago, Chicago, Illinois, United States of America; Clermont Université, France

## Abstract

Sterol Response Element Binding Protein 2 (SREBP2) transcription factor is a master regulator of cholesterol homeostasis. Treatment with statins, inhibitors of cholesterol synthesis, activates intestinal SREBP2, which may hinder their cholesterol-lowering effects. Overactivation of SREBP2 in mouse liver was shown to have no effect on plasma cholesterol. However, the influence of activating intestinal SREBP2 on plasma cholesterol is not known. We have generated a novel transgenic mouse model with intestine specific overexpression of active SREBP2 (ISR2) driven by villin promoter. ISR2 mice showed overexpression of active SREBP2 specifically in the intestine. Microarray analysis of jejunal RNA from ISR2 mice showed a significant increase in genes involved in fatty acid and cholesterol synthesis. Cholesterol and triglyceride (TG) in jejunum and liver (mg/g protein) were significantly increased in ISR2 vs wild type mice. Serum Cholesterol was significantly increased in VLDL and LDL fractions whereas the level of serum triglycerides was decreased in ISR2 vs wild type mice. In conclusion, activation of intestinal SREBP2 alone seems to be sufficient to increase plasma cholesterol, highlighting the essential role of intestine in maintaining cholesterol homeostasis in the body.

## Introduction

Elevated cholesterol level in the plasma is a major risk factor for atherosclerosis and coronary heart diseases [1]. Cholesterol turnover in the body is highly dynamic involving influx and efflux processes across plasma membrane, intracellular trafficking and conversion to bile acids, de novo synthesis and intestinal absorption [1], [2]. These processes are tightly regulated to maintain normal homeostasis in the body providing sufficient supplies and preventing excess of cholesterol [2]. With respect to regulatory mechanisms, the Sterol Response Element Binding Proteins (SREBPs) have been shown to be central regulators of cholesterol and lipid homeostasis [3], [4]. The SREBPs belong to a basic helix-loop-helix leucine zipper (bHLH-zip) family of transcription factors that are present in the endoplasmic reticulum as precursor transmembrane polypeptides associated with multi-protein complex that senses the level of cellular cholesterol [4]. Cellular cholesterol depletion induces the translocation of SREBP precursor to the Golgi apparatus, where the NH2-terminus of ∼460 amino acids is then cleaved in a multistep process and released as an active soluble transcription factor [3]. Three SREBP isoforms have been identified of which SREBP1a and 1c are transcribed from a single gene, whereas, SREBP2 is a product of a distinct gene [3].

The functional roles of SREBPs have been extensively investigated in several cell culture and animal models [5]. These studies were based on either the activation of endogenous SREBPs by cholesterol depletion or the utilization of transgenic approaches in mice by specifically deleting the genes or constitutively overexpressing the NH2-terminus active forms of SREBPs [5]. These investigations yielded important information regarding the genes that are directly modulated by different SREBP isoforms and delineated the metabolic and physiological processes triggered by their activation. For example, studies with liver-specific knockout and liver-specific overexpresison of the active forms of these regulatory proteins showed that SREBP1a and 1c transcription factors preferentially modulate the expression of genes involved in fatty acid synthesis, whereas, SREBP2 mainly regulates the expression of genes involved in cholesterol synthesis and transport [6], [7]. Also, global deletion of both SREBP1a and 1c resulted in embryonic lethality with only ∼15% survival rate. Interestingly, the surviving mice exhibited a compensatory increase in SREBP2 expression [8]. On the other hand, mice with global SREBP2 deletion were not viable with 100% embryonic lethality [9], [10]. These observations indicated that SREBP2 could compensate for the loss of SREBP1 isoforms, whereas, no compensatory mechanisms could rescue the loss of SREBP2.

To understand the physiological and metabolic roles of SREBP2, previous studies mainly focused on the liver [7]. While the liver is a key organ for cholesterol and lipid metabolism in the body, the intestinal functions are also known to be essential for maintaining cholesterol homeostasis [11]. It is, therefore, important to examine the effects of activating SREBP2 specifically in the intestine to determine its effects on the expression of intestinal genes and assess the impact of intestinal SREBP2 on body cholesterol homeostasis. In this regard, treatment with statins, the cholesterol synthesis inhibitors, was recently shown to increase the expression of intestinal SREBP2 demonstrating a compensatory mechanism that may reduce their cholesterol lowering effects [12]. Also, ezetimibe treatment to mice was associated with activation of intestinal SREBP2 [13]. Recent studies provided evidence showing that SREBP2 plays a novel role in many organs including the intestine integrating multiple physiological processes with cholesterol metabolism [14]. For example, SREBP2 has been shown to modulate the expression of the taste receptor T2R in intestinal enteroendocrine cells and the release of the cholecystokinin (CCK) hormone from the intestine [15], [16]. These observations suggest additional roles for intestinal SREBP2 that are not fully understood.

To carefully investigate the influence of SREBP2 on intestinal functions and on body cholesterol homeostasis, we have generated a transgenic mouse model with intestine-specific overexpression of the active SREBP2 (460 amino acid NH2-terminus) driven by the villin promoter to investigate its roles in intestine. Microarray analysis in the jejunum revealed a significant increase in the expression of genes involved in cholesterol and fatty acid synthesis as well as other genes responsible for vitamin transport and circadian rhythm. The levels of plasma cholesterol in the transgenic mice were significantly increased in the LDL and VLDL lipoprotein fractions. These data underscore the emerging roles of intestinal SREBP2 in the maintenance of cholesterol homeostasis and provide a novel mouse model for SREBP2 overexpression in the intestine that complements other models previously described in the liver.

## Materials and Methods

### Transgenic mice with intestine-specific active SREBP2

All animal studies were approved by the animal care committees of the University of Illinois at Chicago and the Jesse Brown VA medical center. A 1380 bp fragment that starts form the ATG codon of the SREBP2 cDNA was amplified from mouse small intestinal RNA using standard PCR methods. This PCR fragment represents the coding region for the first 460 amino acids of the N-terminal of mouse SREBP2. The PCR fragment was then placed downstream of a 9 kb regulatory region of the mouse villin gene (kindly provided by Dr. Sylvie Robine, Institute Curie, Paris, France) [17]. The transgene was engineered to include villin promoter, N-terminal SREBP2 and bovine growth hormone polyadenylation sequence as previously described [18]. Transgenic mice were generated at the Transgenic Production Facility at the University of Illinois at Chicago by pronuclear microinjection of the linear construct including the transgene (villin promoter and the coding region for the N-terminal of SREBP2 gene) into fertilized oocytes of C57BL/6J mice (Jackson Laboratory, Bar Harbor, Maine). Several founders were identified and the colony was established by cross-breeding the transgenic mice with wild-type (WT) C57BL/6J mice obtained from Jackson laboratory. Mice at 8–10 weeks of age were used for all the subsequent genetic and metabolic studies.

### RNA extraction and real time PCR analysis

Total RNA was extracted from tissues utilizing RNeasy Mini Kit (Qiagen) according to the manufacturer's instructions. RNA was treated with DNase I to eliminate genomic DNA contamination. Equal amounts of RNA from different samples were reverse transcribed and amplified in one step reaction utilizing Brilliant SYBR Green QRT-PCR Master Mix Kit (Stratagene). Primers used for the current studies are listed in Supporting Information (**Table S1)**.

The results was expressed as a ratio of 2**^ΔCt(target)^** gene/2**^ΔCt(internal control)^**, where ΔCt represents the difference between the threshold cycle of amplification of RNA from different experimental groups for target gene and internal control gene (GAPDH).

### Protein extraction and western blotting analysis

Total cellular protein was extracted from intestinal mucosal scrapings as previously described with minor modifications [19]. Small pieces of frozen tissues (Jejunum, Ileum and Colon) were homogenized in homogenization buffer containing: 62.5 mM Tris-HCl (pH 7.8), 7% SDS, 8M Urea, and 10 mM DTT. The homogenates were kept at 65°C for 45 minutes followed by sonication for 10 seconds and then incubated at 95°C for 5 min. The samples were then centrifuged at 13,000 RPM for 5 minutes and the clear supernatant representing the total protein lysates were then transferred to a new tube and stored at −80°C. Total protein samples (50–70 µg) were separated by electrophoresis on 10% SDS-polyacrylamide gels and then subjected to western blotting using rabbit anti N-terminal SREBP2 antibodies (Abcam, Cambridge, MA) and mouse anti-villin antibodies (Abcam).

### Immunofluorescence analysis

Sections (5–10 µm thickness) were cut from snap-frozen tissues embedded in OCT medium using a cryostat and were mounted on the slides and preserved at −80°C until further use. For Oil Red-O staining, frozen sections from both wild type and transgenic mice liver were cut at 10 µm thickness. Sections were air dried and fixed in formalin for 10 minutes, washed with running tap water for 10 minutes and then rinsed with 60% isopropanol. Σεχτιονσ were then incubated with freshly prepared working solution of Oil Red-O (Fisher Scientific) for 15 minutes and then rinsed with 60% isopropanol. Nuclei were stained with alum haematoxylin and rinsed with water. Slides were mounted with glycerol and staining was assessed microscopically. For immunofluorescence studies the sections were fixed with 4% paraformaldehyde in PBS for 20 min at room temperature followed by blocking in PBS containing 5% normal goat serum at room temperature. Sections were then incubated with rabbit anti N-terminal SREBP2 antibodies with a dilution of 1∶100 (Abcam) and mouse anti-villin with a dilution of 1∶100 (Abcam) in the blocking buffer. After PBS washes, the sections were incubated with the secondary antibodies, Alexa Fluor 488-conjugated goat anti-mouse IgG (green), and Alexa Fluor 568-conjugated goat anti-rabbit IgG (red) for 60 min and then washed and mounted with slow-fade DAPI (blue, nuclei) by using coverslips. Microscopy was performed with a 20× oil immersion objective of Zeiss immunofluorescence microscope (Observer Z1) equipped with deconvolution software (AxioVision).

### Lipid extraction and the measurement of cholesterol and triglycerides

Total lipid was extracted from intestinal and hepatic tissues essentially as previously described [20]. Briefly, 100 mg of tissue was homogenized in 1 ml of isopropanol with tissue disperser followed by vigorous shaking for 45 minutes and then centrifuged at 13,000 RPM for 10 minutes. Supernatant from each sample was equally divided into 2 parts, one was used for triglyceride measurement and the other was used for cholesterol measurement after adding Triton-×100. Intestinal and hepatic triglycerides were measured using an enzymatic kit (L Type Triglyceride M, Wako diagnostics) according to the manufacturer's instructions. Total cholesterol and free cholesterol were measured using an enzymatic kit (Cholesterol E and Free Cholesterol, respectively, Wako diagnostics, United states) according to the manufacturer's instructions. The amount of cholesterol esters was calculated as the difference between the amount of free cholesterol and total cholesterol. To assess the levels of cholesterol and triglycerides in the blood, serum was first collected and then samples from three different animals from the same group were pooled together for further analysis. Measurement of serum cholesterol and trigycerides as well as lipoprotein fractionation in serum samples was achieved by FPLC methods and performed by the Mouse Metabolic Phenotyping Center University of Cincinnati.

### Statistical analysis

Results are expressed as means ± SE. Student's t-test or one-way ANOVA were utilized in statistical analysis. P<0.05 was considered statistically significant.

## Results

### Generation of transgenic mice with Intestine-Specific overexpression of SREBP2 (ISR2)

**Figure 1:**
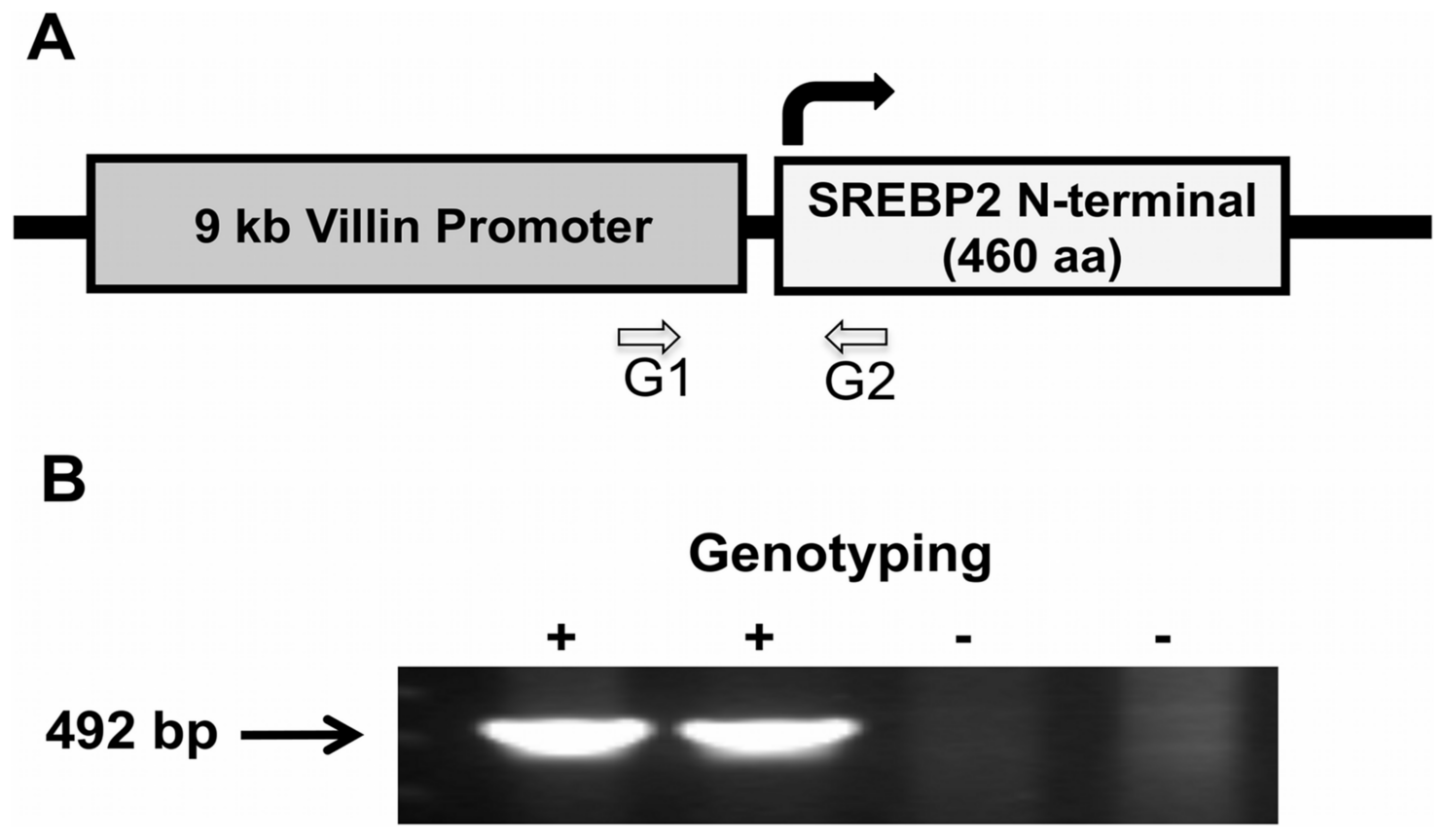
Generation of intestine-specific active SREBP2 mice. The coding sequence for the N-terminal of SREBP2 (representing the active transcription factor) was cloned down-stream of villin promoter. **A**: A schematic representation of the transgene and the location of the G1 and G2 primers used for genotyping and the identification of positive transgenic mice (designated as ISR2) **B**: A representative of genotyping results showing the expected amplified PCR fragment from genomic DNA extracted from ISR2 mice (+) but not their wild type littermates (−).

Recent studies demonstrated putative roles for SREBP2 in regulating different physiological processes including hepatic autophagy and the expression of intestinal taste receptor and the secretion of cholecystokinin (CCK) [15], [16], [21]. To better understand the precise roles of intestinal SREBP2, we initiated the current studies to generate transgenic mice with intestine-specific constitutively active SREBP2. To achieve this goal, we constructed a transgene in which the 9 kb villin promoter was engineered upstream of the coding sequence for the first 460 amino acids (N-terminal) of SREBP2 gene that represent the active transcription factor as previously described [17]. The transgene was then introduced into C57/BL6 mouse and transgenic animals were identified by genotyping PCR using genomic DNA and a pair of primers to amplify a region for the transgene flanking the villin promoter and the N-terminal of the SREBP2 gene. **Figure 1A** shows a schematic diagram representing the transgene. **Figure 1B** shows the results of a PCR experiment depicting that the expected PCR fragment of the transgene is amplified from DNA extracted from the transgenic mice but not from their wild type littermates. These transgenic animals are designated as ISR2 mice.

### SREBP2 N-terminal transgene is specifically expressed in the gastrointestinal (GI) tract

**Figure 2:**
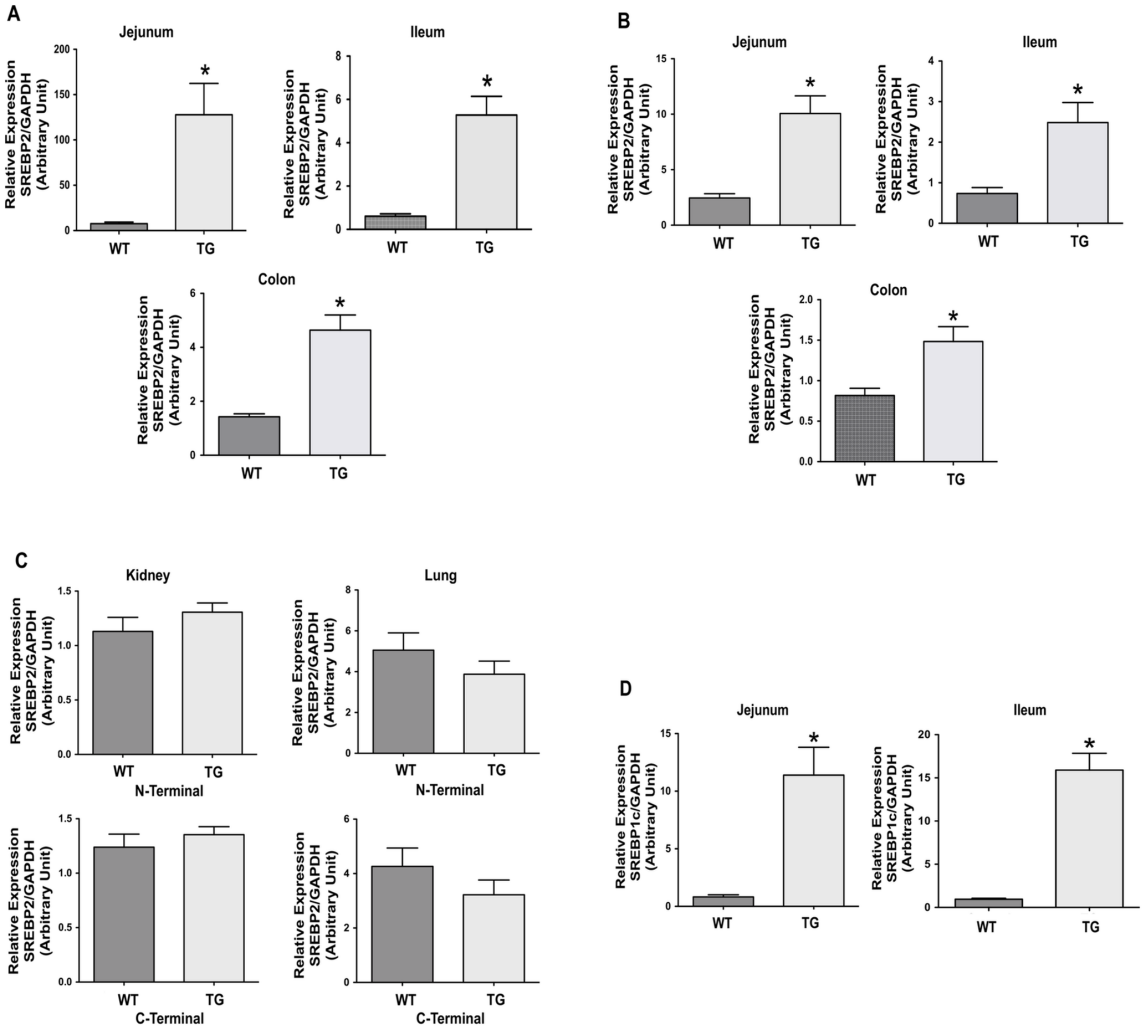
Intestine-specific overexpression of active SREBP2. Total RNA was extracted from different tissues from ISR2 mice (TG) and their wild type littermates (WT) and the expression of SREBP2 was then assessed by real time PCR using gene specific primers. **A**: The expression of active SREBP2 transgene evaluated using a set of primers specific for the N-terminal of SREBP2 mRNA in the jejunum, ileum and colon. **B**: The expression of endogenous SREBP2 mRNA in jejunum, ileum and colon assessed using primers specific for the C-terminal of the SREBP2 gene. **C**: The expression of SREBP2 in the kidney and lung utilizing N- and C-terminal specific primers. **D**: The expression of SREBP1c in jejunum and ileum of ISR2 and WT mice. The presented data represent the expression of respective gene relative to the expression of GAPDH, used as an internal control. The results are expressed as arbitrary unit (A.U.) and represent Mean ± SE using 10–12 animals of each group. * P<0.05 as compared to WT mice.

To determine the expression profile of the N-terminal SREBP2 transgene, we performed real time PCR using total RNA extracted from jejunum, ileum and colon of ISR2 mice and their wild type (WT) littermates. As shown in Figure 2A, the N-terminal segment of SERBP2 is significantly increased in all the regions of the GI tract (∼18 fold increase in jejunum, ∼10 fold in the ileum, and ∼3 fold in colon as compared to wild type littermates) in ISR2 mice as compared to WT littermates indicating the intestinal driven expression of the transgene. We next examined the expression of endogenous SREBP2 by a set of PCR primers that specifically target the C-terminal of the SREBP2 gene. As shown in Figure 2B, the C-terminal expression of SREBP2 was significantly increased in all regions of the GI tract, however, to a lesser extent as compared to the N-terminal of SREBP2. To assess the tissue specificity of the transgene expression, we examined the expression of SREBP2 mRNA in organs other than the intestine. As depicted in Figure 2C, SREBP2 expression was not significantly altered in the kidney and lung of ISR2 mice as compared to WT mice as judged by both the N-terminal and C-terminal sets of PCR primers. These observations indicate that the transgene is not expressed in extra-intestinal tissue confirming the intestine-specific expression of villin promoter-derived active SREBP2. Also, the expression of the other isoform SREBP1c was significantly increased in jejunum and ileum of ISR2 mice as compared to their wild type littermates (Figure 2D). Collectively, these data indicate that the N-terminal SREBP2 transgene is highly expressed in the GI tract, and the transgene stimulated the expression of endogenous SREBP2 and SREBP1c as previously shown [22].

**Figure 3:**
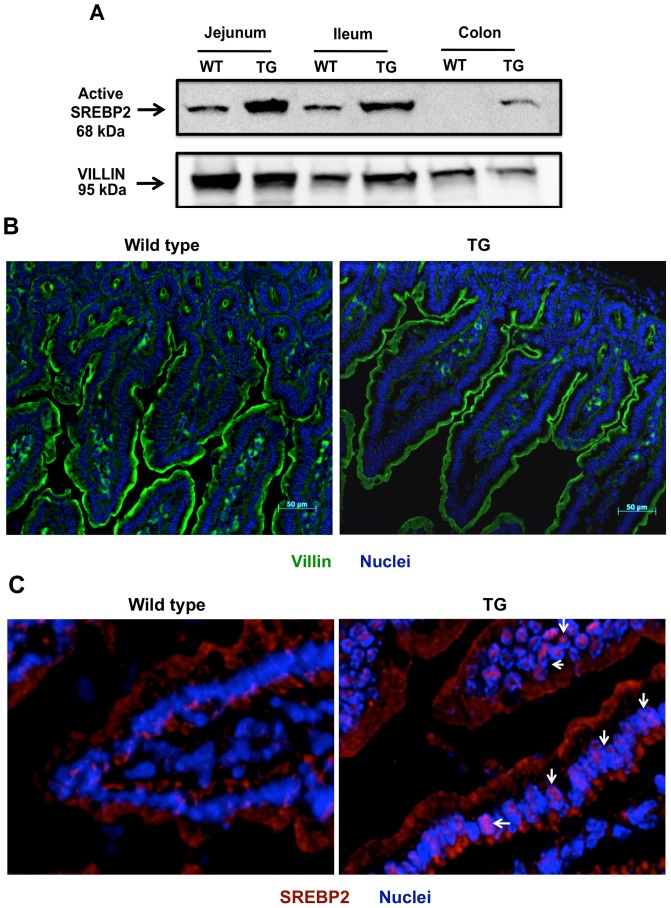
Distribution of active SREBP2 in ISR2 mice. Total protein lysates were prepared form intestinal mucosal scraping as mentioned in Materials and Methods. **A**: A representative blot depicting the bands for active SREBP2 in ISR2 mice and their wild type littermates. Villin was used as a loading control. **B**: Villin staining (green) of the jejunum showing similar epithelial structure in ISR2 and wild type mice. **C**: immuno fluorescence staining of SREBP2 in jejunum of ISR2 and wild type mice. SREBP2 is stained with red and the nuclei with blue. The figure shows predominant cytoplasmic staining in wild type mice and increased colocalization of SREBP2 with the nuclei in ISR2 mice (white arrow).

To confirm the overexpression of the transgene in the intestine, we performed western blot analysis. As shown in **Figure 3A**, the expression of the N-terminal active SREBP2 (∼68 kDa) is significantly increased in jejunum, ileum and colon of ISR2 mice as compared to WT mice. Images presented in **Figure 3B** show villin staining (green) alone in the intestinal epithelia demonstrating no observable alterations in the epithelial structure of intestines from ISR2 mice as compared to WT littermates. **Figure 3C** depicts images from jejunum demonstrating an increase in SREBP2 staining (red) in the nuclei of intestinal epithelial cells in the ISR2 mice. On the other hand, the staining of SREBP2 in the jejunum of WT mice was predominantly observed in the cytoplasm of intestinal epithelial cells. These observations further confirm the results of western blotting showing the activation of SREBP2 in intestinal epithelial cells of ISR2 mice.

### Microarray analysis of gene expression in the jejunum

**Figure 4:**
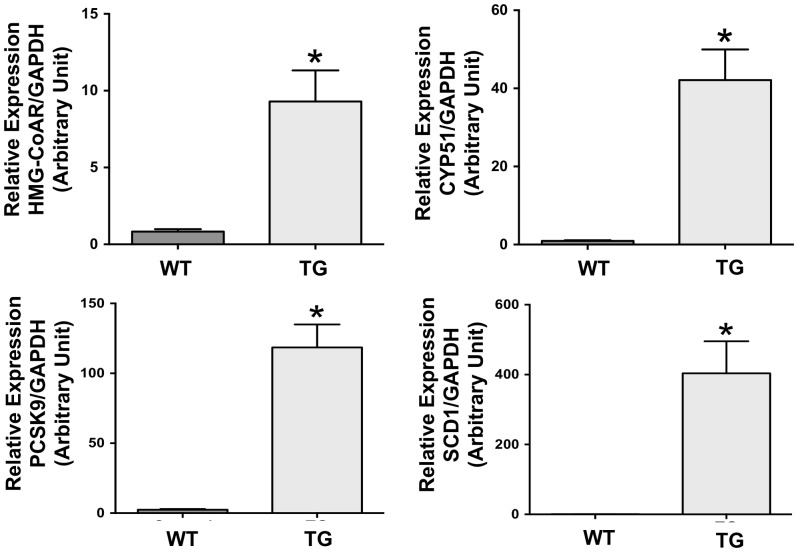
Expression of genes involved in lipid metabolism in ISR2 mice. Expression of HMG-CoA reductase, CYP51, PCSK9 and scd1 were assessed by real time PCR using gene specific primers and total RNA extracted from jejunum of ISR2 mice (TG) and wild type littermates (Control). The results are expressed as arbitrary unit (A.U.) and represent the Mean ± SE using 10–12 animals of each group. * P<0.05 as compared to WT mice.

We next examined the effects of SREBP2 overactivation on gene expression in the intestine. As an initial characterization, we focused on the jejunum since it is the major site for nutrient and cholesterol absorption. We performed gene microarray analysis to identify the genes with altered expression in response to constitutively active SREBP2. The methods for microarray analysis are described in Supporting Information (**Methods S1**). The findings of the microarray analysis show that the expression of several genes is upregulated in ISR2 mice as compared to their wild type littermates and only a few were downregulated. As shown in the **Table 1**, overactivation of SREBP2 in jejunum stimulated the pathways of cholesterol and fatty acid metabolism as well as the expression of other genes involved in various intestinal processes. Notably, the expression of HMG-CoA reductase and CYP51 enzymes involved in cholesterol synthesis was increased as well as the expression of SCD1 and SCD2 enzymes involved in fatty acid synthesis. The expression of LDL receptor and its regulator PCSK9 was also increased. Several members of the SLC gene family including GLUT1 (SLC2A6) and the thiamine transporter 1 (SLC19A2) exhibited a significant increase in their expression in ISR2 mice as compared to WT mice. The results obtained from the microarray analysis were confirmed by real time PCR for selected genes including HMG-CoA reductase, CYP51, PCSK9, and SCD1, as shown in **Figure 4**. The complete list of genes that exhibited significant changes is provided in the Supporting Information (**Table S2**). These data indicate that SREBP2 activation in the jejunum stimulates several pathways that may affect cholesterol homeostasis as well as other processes directly related to intestinal functions.

**Table 1:** Microarray analysis of gene expression in jejunum of ISR2 mice.

Genes	Fold Change
***Cholesterol metabolism***	
3-hydroxy-3-methylglutaryl-Coenzyme A reductase-HMG-CoA R	7
3-hydroxy-3-methylglutaryl-Coenzyme A synthase 1	8
cytochrome P450, family 51	23
squalene epoxidase	16
lanosterol synthase (27)	27
mevalonate kinase (16)	16
mevalonate (diphospho) decarboxylase	16
***Fatty acid metabolism***	
stearoyl-Coenzyme A desaturase 1-Scd1	79
stearoyl-Coenzyme A desaturase 2-Scd2	48
solute carrier family 27 (fatty acid transporter), member 1	2
solute carrier family 27 (fatty acid transporter), member 4	−2
acyl-CoA synthetase long-chain family member 4	11
acyl-CoA synthetase long-chain family member 3	6
ATP citrate lyase	10
***Others***	
low density lipoprotein receptor	7
proprotein convertase subtilisin/kexin type 9-PCSK9	10
Klotho beta	5
low density lipoprotein receptor-related protein 8	3
insulin induced gene 1	10
solute carrier family 2 (facilitated glucose transporter), member 6	3
solute carrier family 19 (thiamine transporter), member 2	3

The increase in gene expression is expressed as a fold change in ISR2 mice as compared to WT mice.

### Lipid content in the jejunum and plasma cholesterol are elevated in ISR2 mice

**Figure 5:**
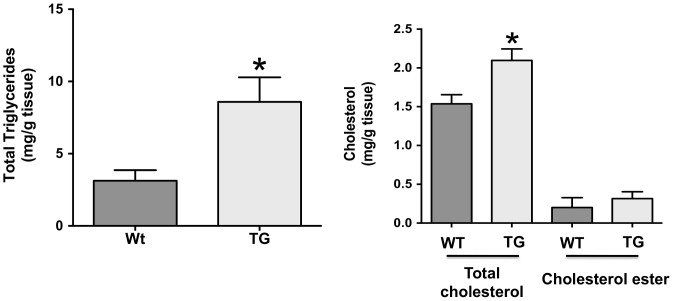
The levels of triglycerides and cholesterol in the jejunum of ISR2 mice. Total lipids were extracted from jejnunal mucosal scrapings from ISR2 (TG) and wild type (WT) mice and the levels of triglycerides, total cholesterol and cholesterol ester were measured as described in Materials and Methods. Data are presented as mg lipid/g of tissues and expressed as Mean ± SE from 8 mice per group. * P<0.05 as compared to WT.

**Figure 6:**
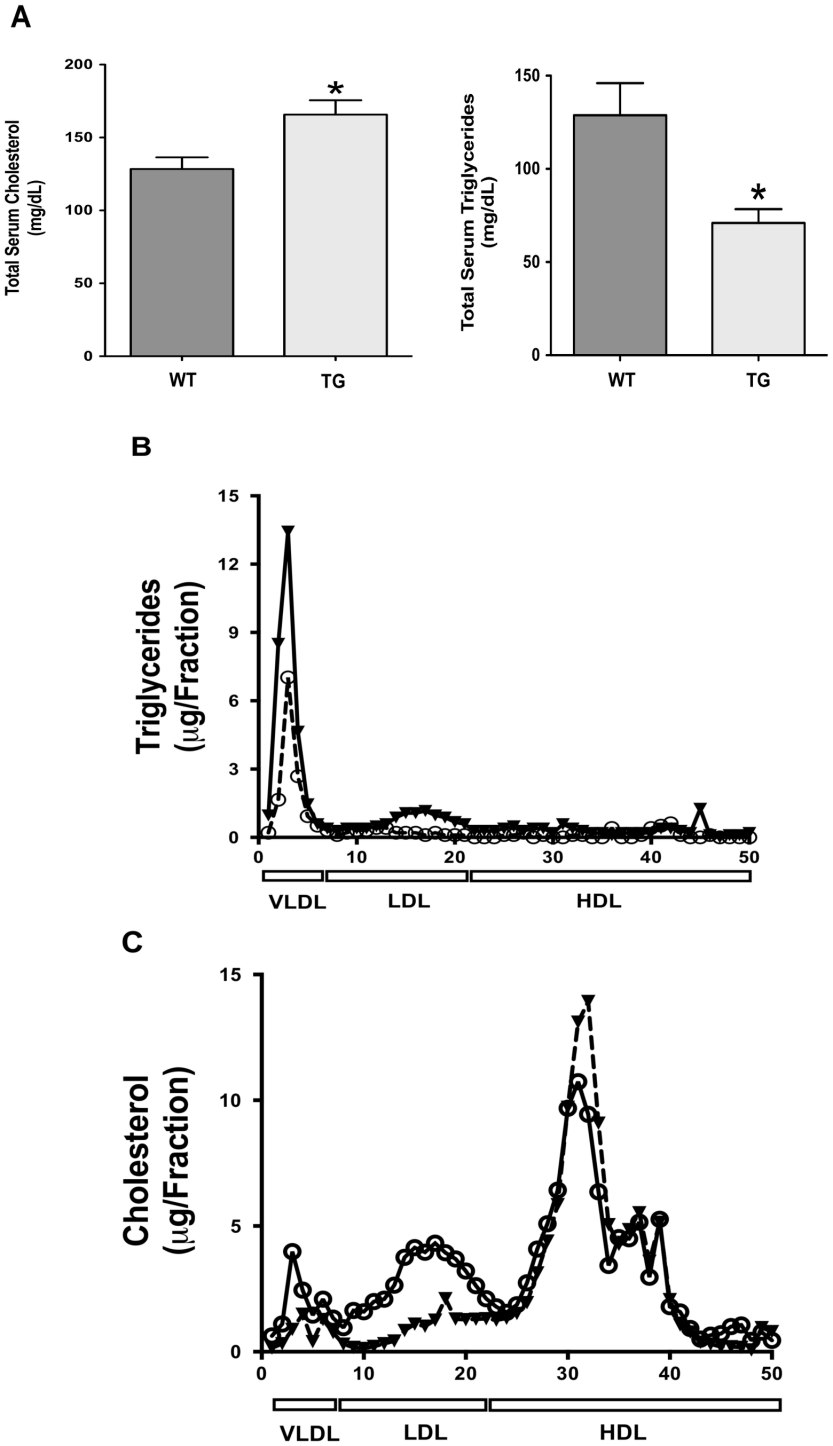
Serum levels of cholesterol and triglycerides in ISR2 mice. **A**: Serum was collected from ISR2 (TG) and wild type (WT) mice. The levels of cholesterol and triglycerides were measured as described in Materials and Methods. The data are expressed as mg/dl and represent the Mean ± SE from 4 animals per group. * P<0.05 as compared to WT. **B**: Triglyceride levels were measured in different lipoprotein fractions prepared from ISR2 transgenic mice and their wild type littermates. The figure shows representative data showing a decrease in triglycerides of the VLDL from the ISR2 (open circles) mice as compared to their wild type littermates (closed triangles). Serum samples from three different ISR2 transgenic mice and three wild type littermates were pooled into one sample and lipoproteins were then obtained by the method of FPLC. **C**: Serum was collected from ISR2 (open circles) and wild type mice (closed triangles) for a total of 12 animals per group, and three animals were pooled into one aliquot for subsequent cholesterol measurement. Cholesterol levels were assessed in different lipoprotein fractions by the FPLC methods. The levels of cholesterol in different lipoprotein fractions are expressed as µg/fraction.

The transgenic ISR2 mice appear healthy and similar to their wild type littermates (up to 12 weeks of age). There is a trend for an increase in the levels of blood glucose in ISR2 mice as shown in **Table 2**. However, the increase is not significant as compared to their wild-type littermates. Also, the ratio of liver to body weight is significantly increased, albeit modestly, in the transgenic mice as compared to wild-type animals suggesting an increase in hepatic lipids. Also, the length of the intestine of the transgenic mice is significantly longer than the wild type mice also suggesting lipid accumulation. We next investigated the effects of overexpressing active SREBP2 in the intestine on lipid content in jejunal mucosa. **Figure 5** shows that the triglycerides and total cholesterol content was significantly increased in the jejunum of ISR2 mice with no alteration in the levels of cholesterol esters. These findings are consistent with the upregulation in the enzymes involved in cholesterol and fatty acid synthesis. We next investigated the level of cholesterol in the blood. As shown in **Figure 6A**, the level of plasma cholesterol was significantly elevated. Surprisingly, the level of serum triglycerides was significantly decreased (**Figure 6A**) despite the fact that intestinal fatty acid synthesis and triglycerides content were increased. The decrease in serum triglycerides was associated with a reduction in their levels in the VLDL fractions as shown in **Figure 6B**. The elevation of serum cholesterol was associated with an increase in total cholesterol associated with the VLDL (5.3±1.3 µg in wild type vs 13±1.2 µg in ISR2 mice, P<0.05) and LDL fractions (12.9±3.9 µg in wild type vs 42.6±1.8 µg in ISR2 mice, P<0.005), whereas there was no significant difference in the levels of cholesterol in the HDL fractions (100.6±2.3 µg in wild type vs 95.5±5.3 µg in ISR2 mice) as assessed by investigating lipoprotein profile depicted in **Figure 6C**. These findings demonstrate that the increase in cholesterol and fatty acid synthesis in the intestine is sufficient to increase serum cholesterol in the VLDL and LDL fractions.

**Table 2:** Phenotypic characterization of ISR2 mice.

	CT	ISR2
Body weight (g)	20.38±0.62	21.23±1.11
Liver weight (g)	0.90±0.05	1.07±0.08
Liver weight/Body weight (g)	0.044±0.001	0.050±0.001*
Intestine weight (g)	1.57±0.06	1.83±0.10
Intestine length (cm)	33.13±0.47	37.12±0.76*
Intestine weight/Intestine length (cm)	0.047±0.001	0.049±0.002
Blood glucose (mg/dL)	224.7±25.17	282±34.94

P<0.05.

### Hepatic lipid content and gene expression in ISR2 mice

**Figure 7:**
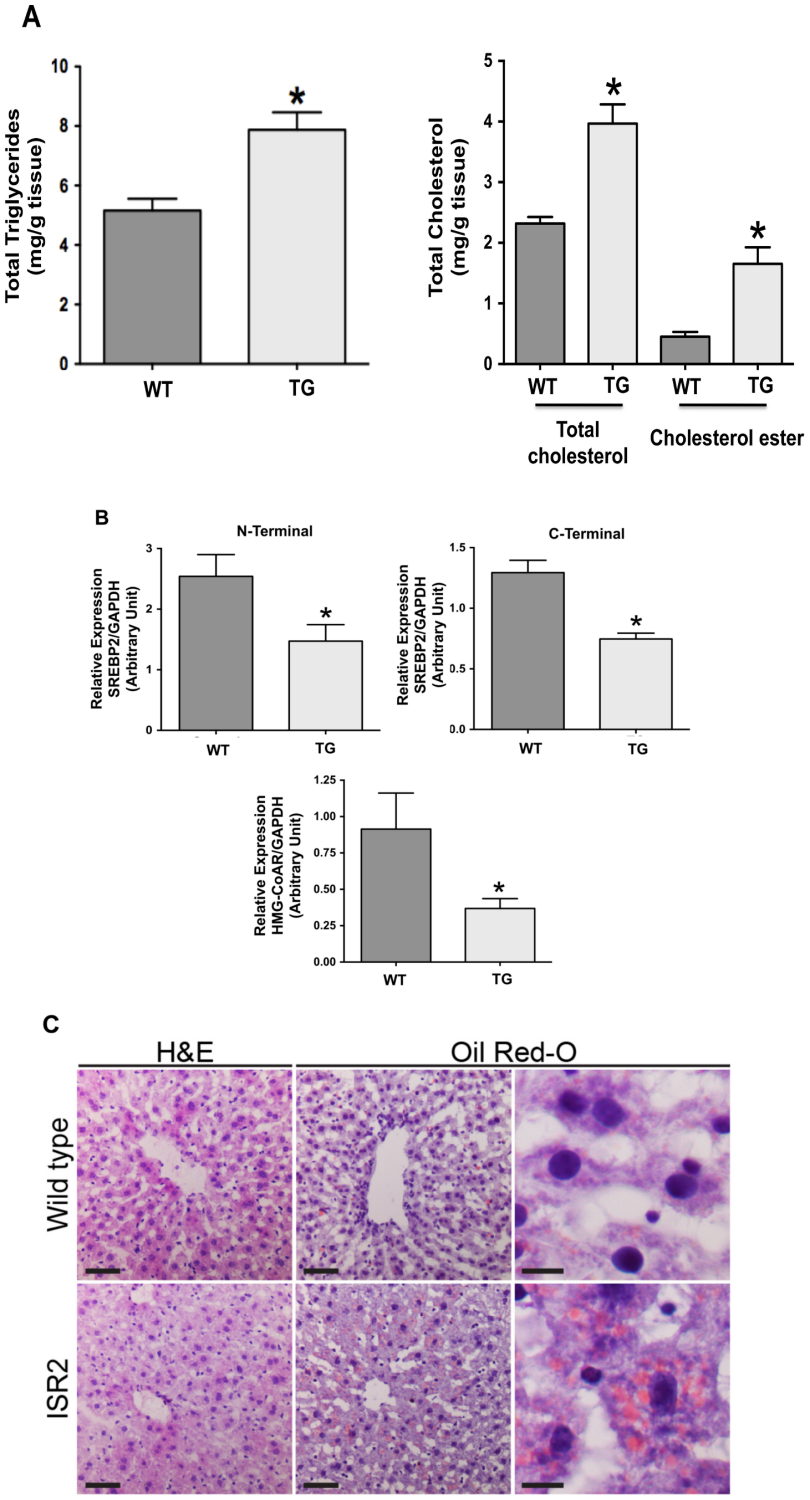
Lipid content in the liver and hepatic gene expression. Total lipids were extracted from livers harvested from ISR2 (TG) and wild type (WT) mice. **A**: The levels of triglycerides, total cholesterol and cholesterol ester are measured in the liver as described in Materials and Methods. The data are expressed as mg lipid/g of hepatic tissue and represent Mean ± SE from 4 animals per group. * P<0.05 as compared to WT. **B**: Total RNA was extracted from livers of ISR2 mice (TG) and wild type mice (control) and the relative expression of SREBP2 was assessed by real time PCR using N- and C-terminal specific primers. The relative expression of the HMG-CoA reductase was also evaluated in the same samples and GAPDH was amplified and used as internal control. The results are expressed as arbitrary unit (A.U.) and represent Mean ± SE of 10–12 animals from each group. * P<0.05 as compared to control mice. **C**: Liver sections from wild type and ISR2 mice stained with hematoxylin and eosin (H&E) at low power (left column) showing the central vein in the center of each image (scale bar = 50 µM). Liver sections were also stained with oil red-O and counterstained with hematoxylin. Low power examination (center column) demonstrated a significant increase in oil red-O staining within the cytoplasm of hepatocytes from ISR2 transgenic mice relative to wild type mice (scale bar = 50 µM). Higher power examination (right column) confirmed the presence of multiple lipid droplets within the cytoplasm of hepatocytes from ISR2 transgenic mice (scale bar = 10 µM).

Since VLDL and LDL cholesterol levels were increased in ISR2 mice, we next investigated lipid content in the liver. As shown in **Figure 7A** the amount of triglycerides in the liver was significantly increased in ISR2 mice as compared to their wild type littermates. Similarly, hepatic total cholesterol and cholesterol ester levels were significantly increased in ISR2 mice indicating lipid overload in the liver as a result of intestinal activation of SREBP2. We next examined the pathways that are triggered by an increase in the level of cholesterol in the liver. As depicted in **Figure 7B**, the increase in hepatic lipid content caused a significant decrease in SREBP2 expression (as assessed by both the N-terminal, representing the transgene, and the C-terminal specific primers) and a subsequent decline in the expression of its target genes HMG-CoA reductase (**Figure 7B**). The expression of SREBP1c in the liver, however, was not significantly altered in ISR2 mice as compared to their wild type littermates (2.1±0.3 arbitrary unit in wild type vs 2.9±0.3 arbitrary unit in ISR2 mice). Histological examination of hepatic sections stained with hematoxylin and eosin (H&E) depicted in **Figure 7C** shows that hepatic architecture, including central veins and hepatocyte cords are unaffected in ISR2 mice. Portal areas were also intact, and there was no inflammatory infiltrate or fibrosis. Hepatocytes of ISR2 transgenic mice were slightly enlarged, in part due to cytoplasmic expansion by clear round spaces within the cytoplasm. The latter also explain the weaker eosin staining of liver sections from ISR2 transgenic animals, relative to wild type mice. To determine if these spaces contained lipid, frozen sections were stained with oil red-O and counterstained with hematoxylin. As shown in **Figure 7C**, there is a significant increase in oil red-O staining within the cytoplasm of hepatocytes from ISR2 transgenic mice relative to wild type mice. Higher power examination confirmed the presence of multiple lipid droplets within the cytoplasm of hepatocytes from ISR2 transgenic mice. Collectively, these findings clearly indicate that livers of ISR2 mice fed with normal chow diet develop mild steatosis and exhibit a normal SREBP2-mediated adaptive response to high levels of cholesterol in the liver. Furthermore, these data also show that active SREBP2 transgene (assessed by the N-terminal PCR) was not expressed in the liver of ISR2 mice further confirming its specific intestinal expression.

## Discussion

Our studies present a novel transgenic mouse model in which SREBP2 transcription factor is constitutively active in intestinal epithelial cells. It was previously shown that liver-specific overactivation of SREBP2 in mice was not associated with an increase in plasma cholesterol [7]. Our data, however, show that the intestine-specific overactivation of SREBP2 was sufficient to cause a significant elevation in plasma cholesterol levels in the VLDL and LDL fractions. Our findings further demonstrated that the constitutive activation of SREBP2 in the intestine caused an increase in the expression of genes including those involved in cholesterol and fatty acid synthesis. The increase in intestinal gene expression was associated with an elevation in tissue levels of cholesterol and triglycerides both in the liver and intestine indicating an increase in lipid synthesis and/or absorption.

The phosphoenolpyruvate carboxykinase (PEPCK) promoter was previously used to drive liver-specific expression of the first 468 aa of SREBP2 that represent the N-terminal active transcription factor [7]. In the current studies, we have utilized the 9 kb villin promoter to specifically overexpress the 460 aa N-terminal fragment of SREBP2 in the intestine. In such a system, SREBP2-mediated pathway in the intestine is constitutively active and is not suppressed by increased levels of cellular cholesterol as occurs under normal conditions. The intestinal overexpression of SREBP2 by the villin promoter was achieved in the C57/BL6 strain of mice and the positive transgenic animals were designated as ISR2 mice. The transgenic animals appeared healthy and fertile and the transgene was transmitted to offspring according to Mendel's laws. Similar to mice with hepatic overexpression of SREBP2 [7], no apparent phenotype was observed in ISR2 mice with regard to body weight and fat distribution as compared to their wild type (WT) littermates up to 12 weeks of age. There was, however, a significant increase in liver/body weight ratio as well as intestinal length of ISR2 mice as compared to wild type mice suggesting lipid accumulation in these tissues.

The expression of the transgene in ISR2 mice was assessed using primers specific for the 5′ region of the mRNA encoding for the N-terminal of the SREBP2. Our data clearly showed that the transgene was specifically over-expressed in the small intestine and the colon but not in other tissues such as liver, lung and kidney. Immunofluorescence analysis in the jejunum confirmed the data obtained by PCR and western blotting showing an enhanced staining for SREBP2 along the length of the villus-crypt axis with increased co-localization in the nuclei indicating activation of the transcription factor. In addition, there was no apparent difference in the villus structure between ISR2 mice and their wild type littermates. The expression of the transgene was different along the small intestine with high levels in the jejunum followed by the ileum and colon. This pattern may be due to regional difference in the activity of the villin promoter. This notion is supported by the fact that the protein expression of endogenous villin as judged by western blotting showed high abundance in jejunum followed by ileum and colon. Western blotting analysis further confirmed the results obtained by PCR regarding the expression of the transgene along the GI tract. It is noted that the endogenous active SREBP2 was detected in both jejunum and ileum but not colon. This is inconsistent with previous studies showing more active nuclear SREBP2 in the nuclear extracts from colon and ileum as compared to jejunum [13]. The fact that our data were collected using total protein lysates but not nuclear extracts may explain the discrepancy between our current results and the previously published findings. In the current studies, total protein lysates rather than nuclear extract were used, as the aim was to only confirm the overexpression of active SREBP2 in ISR2 transgenic mice. We also evaluated the expression of endogenous SREBP2 in different tissues utilizing PCR primers that are specific to the 3′ region of SREBP2 corresponding to the C-terminal of the protein. It appeared that the expression of the endogenous SREBP2 was also increased in all regions of the GI tract but to lower levels as compared to that driven by villin promoter (5 fold increased in C-terminal expression vs 18 fold increase in the N-terminal expression). Both the levels of the N-terminal and the C-terminal were unaltered in extraintestinal tissues such as lung and kidney. The expression of SREBP1c was also significantly increased in the GI tract but not in the liver further suggesting that the activation of SREBP2 also stimulates the expression of SREBP1c as previously suggested [22].

The overexpression of SREBP2 in liver and pancreatic β-cells increases the cellular levels of cholesterol and triglycerides in the liver and pancreas, respectively [7], [23]. Our data also showed that overactivation of SREBP2 in mouse intestine also caused a significant increase in tissue cholesterol and triglycerides in the jejunum. This increase may be attributed to elevated rates of absorption and/or synthesis. Our data clearly showed a significant increase in the expression of enzymes involved in cholesterol synthesis including HMG-CoA reductase and CYP51 as well as enzymes responsible for fatty acid synthesis such as SCD1 and 2 were significantly increased in the jejunum of ISR2 mice. However, the mRNA expression of the NPC1L1 protein responsible for cholesterol absorption was not altered in ISR2 mice. This observation is intriguing in light of previous studies from our laboratories and others demonstrating the presence of sterol response element (SRE) in the human NPC1L1 promoter and showing that active SREBP2 stimulated the promoter activity of human NPC1L1 gene [24]–[26]. It is possible that the increase in cellular levels of cholesterol observed in ISR2 mice stimulated other compensatory pathways in the intestine that counteracted the SREBP2-mediated stimulation in NPC1L1 promoter activity. In this regard, the expression of intestinal proprotein convertase subtillisin/kexin type 9 (PCSK9) expression, a negative regulator of LDL receptor protein and a stimulator of NPC1L1 protein expression, was shown to be significantly increased as judged by our microarray and PCR analysis. This is an important observation in light of recent report demonstrating the intestinal roles of this soluble regulatory protein in the modulation of lipid absorption, synthesis and secretion in the intestine [27]. The observation that NPC1L1 expression is not altered in ISR2 mice despite the fact that SREBP2 is constitutively active and that PCSK9 expression is elevated will be further investigated in future studies.

High levels of free cholesterol in the jejunal mucosa of ISR2 mice were not associated with any significant change in the level of cholesterol ester. This may indicate an overwhelming increase in the loading pathways of free cholesterol (synthesis and/or absorption) to a level that exceeds the capacity of cholesterol esterification pathways mediated by ACAT-2 in the intestine. It is also possible that cholesterol esters did not accumulate in the intestinal epithelial cells due to enhanced secretion of esterified cholesterol from the intestine of ISR2 mice. Indeed, the elevated levels of intestinal cholesterol and triglycerides were associated with an increase in their hepatic levels suggesting that the stimulation of intestinal lipid synthesis and/or absorption caused an overload of lipid in the liver. Although the levels of lipids were increased in the lever, the architecture of the hepatic tissues remained, however, intact and no inflammatory infiltration was observed in livers of ISR2 mice. The Oil Red-O staining confirmed the accumulation of lipids in the hepatocytes and indicated the presence of mild steatosis when mice are fed regular chow diet. It will be interesting in future studies to examine the possible development of steatohepatitis similar to the Non Alcoholic Steatoheptitis (NASH) when ISR2 mice are challenged with high fat diet. The increase in hepatic load triggered normal responses in the liver as judged by the decrease in SREBP2 expression and its target genes [28]. Despite the normal hepatic response to high levels of cholesterol, plasma cholesterol associated with the VLDL and LDL fractions was significantly increased in ISR2 mice as compared to their wild type littermates. It is interesting to note that the levels of plasma triglycerides were significantly decreased in ISR2 mice. Ongoing studies are investigating the basis for the observed decrease in the levels of serum triglycerides in ISR2 mice.

Recent studies demonstrated broader roles for SREBP2 beyond controlling the processes of cholesterol homeostasis [14]. In this regard, SREBP2 was recently shown to modulate the expression of the taste receptor (T2R) in intestinal enteroendocrine cells and the process of cholecystokinin (CCK) secretion [15], [16]. Furthermore, recent evidence suggested a role for SREBP2 in the process of hepatic autophagy [21]. To take advantage of the current model, we have performed a microarray analysis on gene expression in the jejunum to examine the global effect of SREBP2 overactivation on gene expression in the intestine. Our microarray data also unraveled novel findings indicating an increase in the expression of intestinal transport proteins involved in glucose and thiamine absorption. These observations indicate that SREBP2 in not only involved in regulating the expression of intestinal taste receptor and CCK secretion, but it is also involved in the processes of nutrient transport such as glucose and vitamin B1.

Our current data presented herein described a novel model of intestine-specific overactivation of SREBP2 in mice that complement previous models of liver-specific alterations in SREBP2 expression [7], [23]. Our findings indicate that the constitutive overactivation of intestinal SREBP2 alone was sufficient to increase plasma cholesterol associated with VLDL and LDL fractions and stimulate intestinal genes of lipid metabolism as well as genes of vitamin B1 and glucose transport. This animal model is quintessential for exploring the intestinal roles of SREBP2 transcription factor and investigating the contribution of intestinal processes to body cholesterol homeostasis under normal conditions and in response to different types of diet.

## Supporting Information

Methods S1 **Methods for RNA microarray.**
(DOCX)

Table S1 **Sequences for PCR primers.**
(DOCX)

Table S2 **Altered gene expression in the jejunum of ISR2 mice (microarray analysis).**
(PDF)
