# Supplementary material for: Overactivation of Intestinal SREBP2 in Mice Increases Serum Cholesterol
Source: PLoS One. 2014 Jan 20;9(1):e84221. doi: 10.1371/journal.pone.0084221 (PMC3896331; doi:10.1371/journal.pone.0084221)
Supplement: Methods S1 — Methods for RNA microarray. (DOCX) [file pone.0084221.s001.docx]

**Methods S2.**

**Microarray analysis**. Total RNA was extracted from the jejunum of the ISR2 (TG) mice and their wild type (CT) littermates. RNA from three different animals was pooled as one sample. Three samples from the transgenic animals and three from the wild type littermates were then used for microarray analysis that was performed in the Core Gene Facility at the University of Illinois at Chicago. Samples were labeled and hybridized to Affymetrix Mouse Gene 1.0 ST Array according to WT Sense target labeling protocol recommended by (Affymetrix). Each image was analyzed for the following quality metrics: total background, raw noise (Q), average signal present, signal intensity of species-specific house-keeping genes, 3’/5’ signal ratio of house-keeping genes, relative signal intensities of labeling controls, and absolute signal intensities of hybridization controls. All hybridizations passed according to indicated quality criteria. Data was analyzed in Partek Genomics statistical package from Partek, Inc. Hybridization signal intensities were normalized by quantiles and summarized using the Robust Multi-array Average (RMA) [1[ ANOVA test were used to calculate significance of the differential expression. Raw p-values were corrected for False Discovery Rate (FDR) according to step-up or Benjamini-Hochberg procedure [2]. Differentially expressed transcripts were annotated according to Affymetrix ‘NetAffx Analysis Center. Data were submitted to Gene Expression Omnibus with accession number GSE51736.

**References**

1. Irizarry RA, Hobbs B, Collin F, Beazer-Barclay YD, Antonellis KJ, Scherf U, Speed TP (2003) Exploration, normalization, and summaries of high density oligonucleotide array probe level data. Biostatistics 4: 249-64.
2. Benjamini Y, Hochberg Y (1995) Controlling the false discovery rate: a practical and powerful approach to multiple testing. Journal of the Royal Statistical Society Series B: Statistical Methodology 57: 289–300.
