# Supplementary material for: Overactivation of Intestinal SREBP2 in Mice Increases Serum Cholesterol
Source: PLoS One. 2014 Jan 20;9(1):e84221. doi: 10.1371/journal.pone.0084221 (PMC3896331; doi:10.1371/journal.pone.0084221)
Supplement: Table S1 — Sequences for PCR primers. (DOCX) [file pone.0084221.s002.docx]

#### Table S1. Primers used for PCR. BP2N and BP2C are primers specific for N-terminal and C-terminal of SREBP2, respectively. HMG-CoA reductase: 3-Hydroxy-3-methylglutaryl-Coenzyme A reductase; Scd1: Stearoyl-Coenzyme A desaturase 1; Cyp51: Cytochrome P450, family 51.

| **G1 (genomic) Forward** | 5'-ATAGAGGTAGGGAGGTCGAGG-3' |
| --- | --- |
| **G2 (genomic) Reverse** | 5'-CACTGCTGTTGTTGCCACTG-3' |
|  |  |
| **SREBP2 N Forward** | 5'-AGTGGCAACAACAGCAGTGG-3' |
| **SREBP2 N Reverse** | 5'-GCTTTGCACTTGAGGCTGCA-3' |
|  |  |
| **SREBP2 C Forward** | 5'-GACCGCTCTCGAATCCTCTTATGTG-3' |
| **SREBP2 C Reverse** | 5'-GTTTGTAGGTTGGCAGCAGCA-3' |
|  |  |
| **mHmgcr Forward** | 5' - AGAGCGAGTGCATTAGCAAAG -3' |
| **mHmgcr Reverse** | 5' - GATTGCCATTCCACGAGCTAT - 3' |
|  |  |
| **Scd1 Forward** | 5' - TTCTTGCGATACACTCTGGTGC - 3' |
| **Scd1 Reverse** | 5' - CGGGATTGAATGTTCTTGTCGT - 3' |
|  |  |
| **mCyp51 Forward** | 5' - CTTACCTTCTGGGGAGTGATGC - 3' |
| **mCyp51 Reverse** | 5' - AGCCGACCGTAGACTTCTTCT - 3' |
