# Supplementary material for: Overactivation of Intestinal SREBP2 in Mice Increases Serum Cholesterol
Source: PLoS One. 2014 Jan 20;9(1):e84221. doi: 10.1371/journal.pone.0084221 (PMC3896331; doi:10.1371/journal.pone.0084221)
Supplement: Table S2 — Altered gene expression in the jejunum of ISR2 mice (microarray analysis). (PDF) [file pone.0084221.s003.pdf]

Table S2. Microarray analysis in the jejunum of ISR2 mice (TG) vs wild type littermates (CT).

RefSeq	Gene assignment	Gene Symbol	Fold-Change (TG vs. CT)	p-value (TG vs. CT)
	Fatty acid metabolism			
NM_009127	stearoyl-Coenzyme A desaturase 1	Scd1	79.40	0.01
NM_009128	stearoyl-Coenzyme A desaturase 2	Scd2	48.91	0.04
NM_134037	ATP citrate lyase	Acly	10.41	0.03
NM_207625	acyl-CoA synthetase long-chain family member 4	Acsl4	11.15	0.01
NM_028817	acyl-CoA synthetase long-chain family member 3	Acsl3	6.20	0.04
	Cholesterol metabolism			
NM_146006	lanosterol synthase	Lss	27.50	0.02
NM_020010	cytochrome P450, family 51	Cyp51	23.25	0.02
NM_009270	squalene epoxidase	Sqle	16.72	0.02
NM_138656	mevalonate (diphospho) decarboxylase	Mvd	15.86	0.02
NM_028454	transmembrane 7 superfamily member 2	Tm7sf2	14.22	0.02
NM_010191	farnesyl diphosphate farnesyl transferase 1	Fdft1	13.01	0.02
NM_026784	phosphomevalonate kinase	Pmvk	12.94	0.00
NM_010476	hydroxysteroid (17-beta) dehydrogenase 7	Hsd17b7	11.74	0.02
NM_025436	sterol-C4-methyl oxidase-like	Sc4mol	10.77	0.04
NM_023556	mevalonate kinase	Mvk	10.41	0.01
NM_145360	isopentenyl-diphosphate delta isomerase	Idi1	9.79	0.02
NM_134469	farnesyl diphosphate synthetase	Fdps	9.30	0.04
NM_010191	farnesyl diphosphate farnesyl transferase 1	Fdft1	9.03	0.02
NM_145942	3-hydroxy-3-methylglutaryl-Coenzyme A synthase 1	Hmgcs1	8.51	0.02
NM_008255	3-hydroxy-3-methylglutaryl-Coenzyme A reductase	Hmgcr	7.12	0.01
NM_010941	NAD(P) dependent steroid dehydrogenase-like	Nsdhl	6.29	0.01
NM_172769	sterol-C5-desaturase (fungal ERG3, delta-5-desaturase) homolog (S. cerevisae)	Sc5d	3.43	0.03
NM_007898	phenylalkylamine Ca2+ antagonist (emopamil) binding protein	Ebp	5.29	0.02
NM_053272	24-dehydrocholesterol reductase	Dhcr24	3.31	0.01
	Solute carrier family			
NM_172659	solute carrier family 2 (facilitated glucose transporter), member 6	Slc2a6	3.45	0.01
NM_054087	solute carrier family 19 (thiamine transporter), member 2	Slc19a2	3.22	0.03
NM_153150	solute carrier family 25 (mitochondrial carrier, citrate transporter), member 1	Slc25a1	3.18	0.04
NM_008063	solute carrier family 37 (glucose-6-phosphate transporter), member 4	Slc37a4	1.79	0.04
NM_011977	solute carrier family 27 (fatty acid transporter), member 1	Slc27a1	1.77	0.04
NM_011989	solute carrier family 27 (fatty acid transporter), member 4	Slc27a4	-1.57	0.03
	Lipid homeostasis			
NM_011125	phospholipid transfer protein	Pltp	6.74	0.01
NM_133774	StAR-related lipid transfer (START) domain containing 4	Stard4	3.04	0.01
NM_015730	cholinergic receptor, nicotinic, alpha polypeptide 4	Chrna4	55.52	0.01
NM_153565	proprotein convertase subtilisin/kexin type 9	Pcsk9	10.64	0.01
NM_010700	low density lipoprotein receptor	Ldlr	6.67	0.03
NM_031180	klotho beta	Klb	4.92	0.01

NR_033496	low density lipoprotein receptor-related protein 8, apolipoprotein e receptor	Lrp8	3.36	0.02
NM_153526	insulin induced gene 1	Insig1	10.38	0.00
	Miscellaneous			
NM_011147	protein phosphatase with EF hand calcium-binding domain 1	Ppef1	59.16	0.02
NM_178730	transmembrane protease, serine 11f	Tmprss11f	21.43	0.03
NM_015763	lipin 1	Lpin1	16.96	0.00
NM_019811	acyl-CoA synthetase short-chain family member 2	Acss2	12.33	0.02
NM_008615	malic enzyme 1, NADP(+)-dependent, cytosolic	Me1	11.26	0.02
NM_010391	histocompatibility 2, Q region locus 10	H2-Q10	11.24	0.02
NM_001033498	GRAM domain containing 2	Gramd2	7.87	0.04
NM_021557	retinol dehydrogenase 11	Rdh11	7.52	0.02
NM_021462	MAP kinase-interacting serine/threonine kinase 2	Mknk2	6.59	0.03
NM_008685	nuclear factor, erythroid derived 2	Nfe2	5.76	0.02
NM_011921	aldehyde dehydrogenase family 1, subfamily A7	Aldh1a7	5.62	0.03
NM_001177503	RIKEN cDNA 3830431G21 gene	3830431G21Rik	5.50	0.03
NM_001163427	RIKEN cDNA 1700040I03 gene	1700040I03Rik	5.29	0.03
NM_001037928	predicted gene 11992	Gm11992	5.12	0.02
NM_021446	RIKEN cDNA 0610007P14 gene	0610007P14Rik	4.85	0.04
NM_019405	centrin 2	Cetn2	4.02	0.04
NM_008686	nuclear factor, erythroid derived 2,-like 1	Nfe2l1	4.01	0.04
NM_028372	metallo-beta-lactamase domain containing 2	Mblac2	4.00	0.04
NM_001081423	tubulin tyrosine ligase-like family, member 5	Ttll5	3.89	0.03
NM_029730	motile sperm domain containing 2	Mospd2	3.70	0.02
NM_172734	serine/threonine kinase 38 like	Stk38l	3.12	0.03
NM_199308	microtubule associated serine/threonine kinase 3	Mast3	3.09	0.04
NM_029956	methylmalonic aciduria (cobalamin deficiency) type B homolog (human)	Mmab	3.08	0.02
NM_198168	protein phosphatase 2, regulatory subunit B (B56), beta isoform	Ppp2r5b	2.59	0.01
NM_013463	galactosidase, alpha	Gla	2.46	0.04
NM_031843	dipeptidylpeptidase 7	Dpp7	2.42	0.03
NM_181649	coiled-coil domain containing 75	Ccdc75	2.33	0.03
NM_010497	isocitrate dehydrogenase 1 (NADP+), soluble	Idh1	1.93	0.03
NM_001033271	transmembrane protein 55b	Tmem55b	1.77	0.02
NM_010431	hypoxia inducible factor 1, alpha subunit	Hif1a	1.75	0.04
NM_024204	ankyrin repeat domain 22	Ankrd22	1.60	0.04
NM_007764	v-crk sarcoma virus CT10 oncogene homolog (avian)-like	Crkl	1.59	0.03
NM_015774	ERO1-like (S. cerevisiae)	Ero1l	1.47	0.01
NM_021885	tubby candidate gene	Tub	-1.16	0.02
ENSMUST00000057551	stem-loop binding protein	Slbp	-1.22	0.05
NM_010719	lipase, hormone sensitive	Lipe	-1.47	0.02
NM_025700	phosphoglucomutase 1	Pgm1	-1.90	0.05
